# National Trends in Vital Sign Abnormalities at Arrival to the Emergency Department

**DOI:** 10.5811/westjem.58990

**Published:** 2023-05-05

**Authors:** Rama A. Salhi, Margaret Greenwood-Ericksen, Keith E. Kocher

**Affiliations:** * Department of Emergency Medicine, Massachusetts General Hospital, Boston, Massachusetts; † Department of Emergency Medicine, University of New Mexico, Albuquerque, New Mexico; ‡ Department of Psychiatry and Behavioral Sciences, University of New Mexico, Albuquerque, New Mexico; § Department of Emergency Medicine, University of Michigan, Ann Arbor, Missouri

## Abstract

**Introduction:**

Recent reports suggest rising intensity of emergency department (ED) billing practices, sparking concerns that this may represent up-coding. However, it may reflect increasing severity and complexity of care in the ED population. We hypothesize that this in part may be reflected in more severe manifestations of illness as indicated by vital sign abnormalities.

**Methods:**

Using 18 years of data from the National Hospital Ambulatory Medical Care Survey, we conducted a retrospective secondary analysis of adults (>18 years). We assessed standard vital signs using weighted descriptive statistics (heart rate, oxygen saturation, temperature, and systolic blood pressure [SBP]), as well as hypotension and tachycardia. Finally, we evaluated for differing effects stratifying by subpopulations of interest, including age (<65 vs ≥65), payer type, arrival by ambulance, and high-risk diagnoses.

**Results:**

In total there were 418,849 observations representing 1,745,368,303 ED visits. We found only minimal variations in vital signs over the study period: heart rate (median 85, interquartile range [IQR] 74–97); oxygen saturation (median 98, IQR 97–99); temperature (median 98.1, IQR 97.6–98.6); and SBP (median 134, IQR 120–149). Similar results were found among the subpopulations tested. The proportion of visits with hypotension decreased (first/last year difference 0.5% [95% CI 0.2%–0.7%]) while there was no difference in the proportion of patients with tachycardia.

**Conclusions:**

Arrival vital signs in the ED have largely remained unchanged or improved over the most recent 18 years of nationally representative data, even for key subpopulations. Greater intensity in ED billing practices is not explained by changes in arrival vital signs.

## INTRODUCTION

Recently there has been increasing scrutiny of emergency department (ED) billing practices.^1^ A report released by the Office of the Inspector General revealed a 21% increase in the highest reimbursement category between 2001–2010.^2^,^3^ Explanations to account for these trends include concerns about billing at a level of care higher than appropriate for the services rendered, referred to as “up-coding,” greater adoption and integration of electronic health records that enhance billing processes, and changes related to ED clinical practices pressures, especially tied to greater intensity of services as a result of the changing complexity, illness, or clinical instability of patients.^4^,^5^ Understanding the sources of these trends is important given increasing efforts to contain ED expenditures in the setting of rising healthcare costs.

One important hypothesis to consider that may explain rising intensity in ED billing practices is increased severity of illnesses presenting to EDs. Prior work has used claims-based data to explore correlations between coding intensity on claims and a variety of surrogate markers of illness severity, including acuity assignments and billed ED services such as use of procedures or diagnostic testing.^4^ However, these markers are also confounded by temporal and evolving trends in clinical practice rather than differences in the level of illness severities confronted in the ED, limiting their ability to trend increasing clinical acuity over time. Further, while generally felt to be reliable metrics to identify high-resource patients,^6^ acuity assignments remain vulnerable to potential bias related to factors such as physician clinical knowledge, environmental constraints, and patient demographics.^7^

Vital sign measurements provide an alternative approach to measuring severity of illness. Since measurement of vital signs is standard and central to the clinical assessment and treatment of ED patients, they provide a useful objective measure with resistance to the temporal biases that are encountered with other metrics and can act as a proxy for patient severity and acuity of illness.^8^ We examined a nationally representative dataset with longitudinally consistent, data-definition standards to test for differences over time in the vital signs of patients arriving to the ED. Specifically, we hypothesized that increasing severity of illness, as measured by vital sign instability (defined as hypotension (systolic blood pressure [SBP] <90), tachycardia (heart rate >100), or >1 abnormal vital sign), may be correlated with known increased intensity in ED billing practices over time.

## METHODS

We used the most recent 18 years (2001–2018) of the National Hospital Ambulatory Medical Care Survey (NHAMCS) for this analysis. The NHAMCS is an annual, national probability sample of ambulatory visits made to non-federal general and short-stay hospitals in the US, which is conducted by the National Center for Health Statistics. Sample hospitals are randomly assigned to 16 groups that rotate across four-week reporting periods so that each hospital is surveyed about once every 15 months.^9^ Information about ED visits is abstracted from chart review using standard data definitions, including demographics, vital signs, and diagnostic codes.

Because children particularly have varying definitions of abnormal vital signs dependent on age, we excluded 125,518 patients <18 years old. We then calculated and trended weighted descriptive statistics for the following vital signs: heart rate; oxygen saturation; temperature; and SBP. Given non-normal distribution, median and interquartile ranges [IQR] are reported. Respiratory rate, also available in the dataset, was excluded due to significant missingness (>60%). As repeat vital signs are not measured in all patients, we used vital signs on arrival to the ED for this analysis.

To analyze common clinically relevant measures, we also assessed for trends in vital sign instability. This included tachycardia (pulse >100), hypotension (SBP <80), abnormal temperature (temperature <95°F or ≥100.4°F), and hypoxia (SpO_2_ <88%) Finally, we evaluated for the possibility of differing effects across important ED subpopulations that were defined a priori, stratifying by age (<65 vs ≥65), payer (uninsured, private, government), ambulance arrival, and previously described high-risk diagnoses.^10^ High-risk diagnoses were defined as those having greater than 3% inpatient mortality and include the following: pneumonia; congestive heart failure; acute myocardial infarction; stroke; sepsis; gastrointestinal bleed; acute renal failure; and respiratory failure.

We calculated survey-weighted summary statistics for each of the available vital signs, and differences between the first and last year of study were calculated using post-estimation for linear combinations of variables. To assess for trends in clinical instability over time, we completed survey-weighted logistic regressions. Details regarding the methodology used to address annual NHAMCS survey revisions and data collection changes can be found in the manuscript supplement. All analyses were completed in StataSE v17.0 (StataCorp LLC, College Station, TX), and the study was deemed exempt from review by the University of Michigan Institutional Review Board.

## RESULTS

In total there were 418,849 observations representing 1,932,843,890 ED visits from 2001–2018. The median age was 43 years (interquartile range [IQR] 29–60) with 43.1% male (Supplement Table 1). Vital sign trends analyzed revealed minimal variation over the study period (Figure). Heart rate measurements remained stable (median 85, IQR 74–97; yearly median range 84–85). Similar trends were noted in measurements of oxygen saturation (median 98, IQR 97–99; all yearly medians 98), temperature (median 98.1°, IQR 97.6–98.6°; yearly median range 98–98.2°), and SBP (median 134, IQR 120–149; yearly median range 133–135). Finally, among the assessed subpopulations evaluated, we found no difference in vital sign trends over time (Supplement).

We also evaluated for differences in the proportion of ED patients with unstable arrival vital signs but found no evidence of increasing severity. The percentage of hypotensive visits decreased over time, accounting for 1.1% in 2001 to 0.6% in 2018 (difference of −0.5%; 95% confidence interval [CI] −0.2% – −0.7%). In addition, we saw no clear trends in patients presenting with tachycardia, with this proportion being 22.9% in 2001 as compared to 25.1% in 2018 (difference of 2.2%; 95% CI −5.4%–1.0%) (Supplement Figure 2). The proportion of patients presenting with >1 abnormal vital sign was 9.2% in 2001 and 6.7% in 2018 (difference of −2.5%; 95% CI −5.2%–0.3%). When evaluating the odds of presentation with signs of clinical instability over time, we saw there was no change in the likelihood of tachycardia (*P*=0.22) or hypoxia (*P*=0.15) over the study period. For hypotension and abnormal temperature, we noted decreasing odds over time (*P*<0.01 for both measures). Similar trends were noted in all subgroups.

## DISCUSSION

In this nationally representative data of ED visits, we found no indication of increased severity of illness, as measured by initial vital sign abnormalities at time of ED presentation. This trend persisted among subpopulations of interest, including high-risk diagnoses, patients ≥65, ambulance arrival, and publicly insured patients. Similarly, when looking at tachycardia, there was minimal change over the studied period. Notably, there was a decrease in the proportion of hypotensive patients presenting to the ED over the study period, accounting for 1.1% of patients in 2001 to 0.6% in 2018.

Our findings suggest that trends in increasing billing practices are not correlated with increasing vital sign instability. However, while the proportions and central estimates of these results do not suggest overall increases in the severity of illness in the average ED patient, our study years overlap with considerable temporal changes in ED care delivery. These changes include the implementation of electronic health records, which allows for improved capture of clinical elements and thus a higher level of billing, implementation of the Affordable Care Act, and Medicare expansions coupled with increasing numbers of hospital closures, which have resulted in increasing patient volumes with decreased access to local EDs and other venues for acute unscheduled care.^11^ There has also been evolving pressures on EDs to implement more intense and complex care management practices prior to hospitalization or discharge.^12^ This may include increased critical care rendered in the ED as hospital crowding increases.^13^ Additionally, as prehospital care practices and protocols have become increasingly sophisticated, the observed trends in vital signs may be confounded by earlier stabilization of medical conditions prior to presentation to the ED.

## LIMITATIONS

Limitations of this study include the use of a single measure of vital signs rather than serial measures during the ED visit, as well as use of vital sign abnormalities as a surrogate for measuring trends over time in ED acuity and severity of illness. Vital sign changes may be only one potential component of clinical complexity in the ED. Other factors include increasing patient age, greater comorbidities and chronic disease burden, and rising demands on ED evaluations such as higher intensity of diagnostic testing and pressures to avoid hospitalization, which have all been shown to be increasing.^4^,^12^ However, even in the setting of these limitations, vital signs and clinical instability remain an important component of the evaluation of illness severity among patients presenting to the ED.

Further, limitations of the dataset we used include the lack of availability of respiratory rate, which is particularly relevant for cardiopulmonary disorders. That being acknowledged, the remainder of the available vital sign data independently provide important information that contributes the consideration of illness severity. Utilization of this national sample provides estimates that have broad generalizability but may not necessarily reflect trends seen in smaller communities. The limitations are among those previously noted to be inherent in the utilization and interpretation of NHAMCS data.^14^

## CONCLUSION

Vital signs provide an objective, standard measure of patient illness severity that is both clinically relevant and can be trended over time.^8^ When analyzing vital signs as one component of illness severity, we note that they remain largely unchanged or improved, even for key subpopulations. These results, in the context of greater intensity in ED billing practices, do not suggest a correlation with changes in illness severity, specifically as measured by arrival vital signs.

## Supplementary Information



## Figures and Tables

**Figure:**
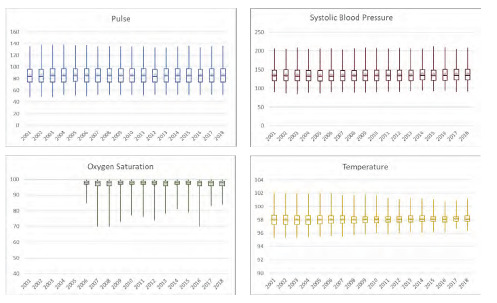
Annual trends in vital sign abnormalities.*,** *Box and whisker plot intervals represent 1st, 25th, 50th, 75th, and 99th percentiles. Circle overlying box and whisker plot represents annual mean. **Reference lines have been demarcated for pulse=100; systolic blood pressure=90; oxygen saturation=88%; temperature=100.4°F.
