# Supplementary material for: National Trends in Vital Sign Abnormalities at Arrival to the Emergency Department
Source: West J Emerg Med. 2023 May 5;24(3):401–4. doi: 10.5811/westjem.58990 (PMC10284526; doi:10.5811/westjem.58990)
Supplement: Supplementary file 1 [file wjem-24-401-S.docx]

**Supplement**

CCS and CCSR Coding Details:

In 2016, NHAMCS switched to ICD10 from 1CD9. As a result, for the subcategory of high-risk diagnoses, the HCUP clinical classification software (CCS)* for ICD-10 was used. High risk diagnoses are as follows:

- Pneumonia (CCS – 122; CCSR – RSP002)
- CHF (CCS – 108; CCSR – CIR019)
- Sepsis (CCS – 2; CCSR – INF002)
- AMI (CCS – 100; CCSR – CIR009)
- Stroke (CCS – 109; CCSR – CIR020)
- Respiratory failure (CCS – 131; CCSR – RSP012)
- GI Bleed (CCS – 153; CCSR – DIG021)
- ARF (CCS – 157; CCSR – GEN002)

* Clinical Classifications Software Refined (CCSR). Healthcare Cost and Utilization Project (HCUP). October 2021. Agency for Healthcare Research and Quality, Rockville, MD. www.hcup-us.ahrq.gov/toolssoftware/ccsr/ccs_refined.jsp.

**Table 1. Summary demographic statistics of patients included in NHAMCS, 2001-2018. (Weighted N=1,745,368,303)**

| Demographics |  | |  | |  |  | |
| --- | --- | --- | --- | --- | --- | --- | --- |
|  | **Overall** | **2001** | | **2018** | | | **Yearly Range** |
|  |  |  | |  | | |  |
| Age, MEDian (iqr) | 43 (29-60) | 41 (29-58) | | 47 (31-63) | | | 41-47 |
| Male, % | 43.1 | 44.6 | | 42.6 | | | 41.8-44.6 |
| Payer type*, % |  |  | |  | | |  |
| *Private* | 37.0 | 37.8 | | 32.7 | | | 32.7-42.0 |
| *Medicare* | 21.9 | 19.2 | | 24.7 | | | 17.9-24.7 |
| *Medicaid* | 23.1 | 12.5 | | 30.4 | | | 12.5-32.8 |
| *Self-pay* | 16.2 | 16.5 | | 10.8 | | | 9.5-20.6 |
|  |  |  | |  | | |  |
| AMBULANCE Arrival**, % | 18.9 | 18.4 (***2003***) | | 19.6 | | | 17.3-20.5 |
| High risk diagnoses, % | 5.5 | 6.0 | | 6.0 | | | 4.3-6.6 |
|  |  |  | |  | | |  |
|  |  |  | |  | | |  |
| Vital signs, Median (IQR) |  |  | |  | | |  |
| Heart Rate | 85 (74-97) | 84 (74-96) | | 85 (74-97) | | | 84-85 |
| Systolic blood pressure | 134 (120-149) | 134 (120-149) | | 135 (122-151) | | | 133-135 |
| Oxygen Saturation | 98 (97-99) | 98 (97-99; ***2006***) | | 98 (96-99) | | | 98-98 |
| Temperature | 98.1 (97.6-98.6) | 98 (97.3-98.7) | | 98.1 (97.8-98.6) | | | 98-98.2 |
|  |  |  | |  | | |  |
|  |  |  | |  | | |  |
| Critical Vital signs, % |  |  | |  | | |  |
| Tachycardia | 23.9 | 22.9 | | 25.1 | | | 22.2-26.7 |
| Hypotension | 0.8 | 1.0 | | 0.6 | | | 0.6-1.2 |

*Prior to 2005, entries only allowed for one payer type. For 2005 and beyond, patients may have multiple payer types.

**Reflects data from 2003-2018 as the measure was not collected in 2001 and 2002.

**Figure 2. Annual trends in proportion of patients presenting with tachycardia or hypotension, % (95% CI)***

**
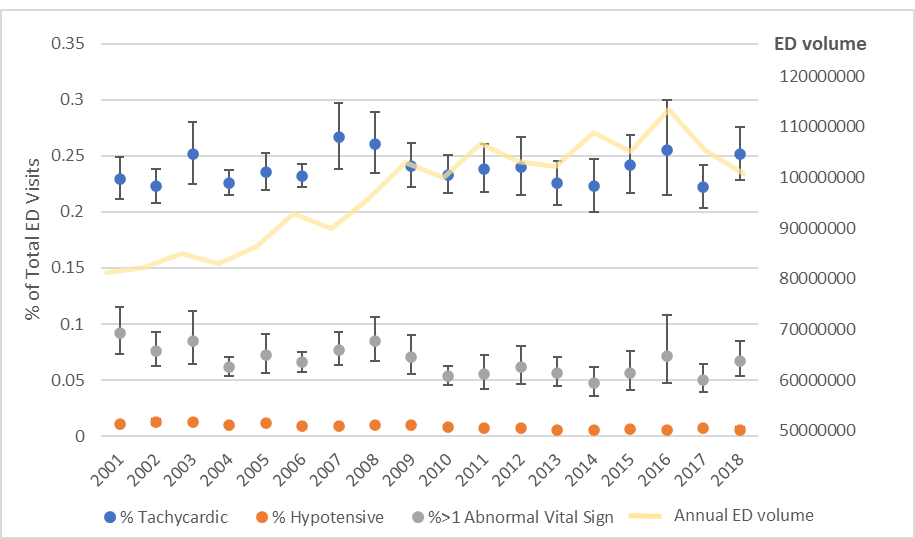
**

*Error bars on hypotension unable to be displayed graphically due to narrow margins. Values presented in Table 2.

**Table 2. Central estimates and 95% CI for patients presented to the ED with tachycardia and hypotension.**

|  | % Tachycardic | 95% CI | | % Hypotensive | 95% CI | |
| --- | --- | --- | --- | --- | --- | --- |
| 2001 | 22.9 | 21.1, | 24.9 | 1.0 | 0.9, | 1.2 |
| 2002 | 22.3 | 20.8, | 23.8 | 1.2 | 1.0, | 1.5 |
| 2003 | 25.1 | 22.5, | 28.0 | 1.2 | 1.1, | 1.4 |
| 2004 | 22.6 | 21.5, | 23.8 | 1.0 | 0.9, | 1.1 |
| 2005 | 23.6 | 22.0, | 25.3 | 1.1 | 1.0, | 1.4 |
| 2006 | 23.2 | 22.2, | 24.3 | 0.9 | 0.8, | 1.1 |
| 2007 | 26.7 | 23.8, | 29.7 | 0.9 | 0.8, | 1.0 |
| 2008 | 26.1 | 23.4, | 28.9 | 1.0 | 0.9, | 1.2 |
| 2009 | 24.1 | 22.2, | 26.2 | 1.0 | 0.8, | 1.1 |
| 2010 | 23.3 | 21.7, | 25.1 | 0.8 | 0.7, | 1.0 |
| 2011 | 23.8 | 21.7, | 26.1 | 0.7 | 0.6, | 0.9 |
| 2012 | 24.0 | 21.5, | 26.7 | 0.7 | 0.5, | 0.9 |
| 2013 | 22.5 | 20.6, | 24.6 | 0.6 | 0.5, | 0.7 |
| 2014 | 22.3 | 20.0, | 24.7 | 0.6 | 0.4, | 0.8 |
| 2015 | 24.1 | 21.6, | 26.8 | 0.6 | 0.5, | 0.8 |
| 2016 | 25.5 | 21.5, | 29.9 | 0.5 | 0.4, | 0.7 |
| 2017 | 22.2 | 20.4, | 24.2 | 0.7 | 0.5, | 0.9 |
| 2018 | 25.1 | 22.8, | 27.5 | 0.6 | 0.4, | 0.8 |

| Demographics |  | |  | |  | |  | | |  | |  |  | |  | |  | |  |
| --- | --- | --- | --- | --- | --- | --- | --- | --- | --- | --- | --- | --- | --- | --- | --- | --- | --- | --- | --- |
|  | **Overall** | **Yearly Range** | | **PNA** | | **CHF** | | **Sepsis** | **CVA** | | **AMI** | | | **Resp Failure** | | **GI Bleed** | | **ARF** | |
|  |  |  | |  | |  | |  |  | |  | | |  | |  | |  | |
| Age, MediaN (IQR) | 67  (52-80) | 64-69 | | 65  (48-79) | | 74  (61-83) | | 70  (54-81) | 71  (58-81) | | 65  (52-78) | | | 66  (53-78) | | 61  (43-77) | | 67  (54-79) | |
| Male, % | 49.0 | 45.8-51.7 | | 46.8 | | 47.8 | | 52.8 | 46.4 | | 55.2 | | | 49.1 | | 49.6 | | 55.2 | |
| Payer type*, % |  |  | |  | |  | |  |  | |  | | |  | |  | |  | |
| *Private* | 36.5 | 23.7-44.2 | | 37.5 | | 32.2 | | 33.6 | 38.4 | | 44.5 | | | 36.4 | | 37.6 | | 32.2 | |
| *Medicare* | 52.5 | 45.7-60.0 | | 48.9 | | 66.4 | | 58.3 | 55.4 | | 46.9 | | | 54.4 | | 42.4 | | 57.7 | |
| *Medicaid* | 19.0 | 9.4-30.4 | | 20.3 | | 18.0 | | 23.2 | 14.9 | | 13.7 | | | 19.8 | | 18.2 | | 24.6 | |
| *Self-pay* | 7.6 | 2.6-12.7 | | 8.9 | | 5.3 | | 5.4 | 6.9 | | 7.7 | | | 9.4 | | 10.0 | | 5.2 | |
| AMBULANCE Arrival**, % | 42.2 | 37.4-46.0 | | 37.3 | | 45.2 | | 60.0 | 53.9 | | 44.6 | | | 71.9 | | 30.2 | | 44.3 | |
|  |  |  | |  | |  | |  |  | |  | | |  | |  | |  | |
| Vital signs, Median (IQR) |  |  | |  | |  | |  |  | |  | | |  | |  | |  | |
| Heart Rate | 88  (75-104) | 87-90 | | 95  (82-110) | | 85  (72-100) | | 102  (86-119) | 78  (68-90) | | 82  (70-98) | | | 102  (82-119) | | 86  (75-99) | | 85  (72-100) | |
| Systolic blood pressure | 136  (118-156) | 133-138 | | 134  (117-151) | | 142  (121-163) | | 120  (101-142) | 151  (132-173) | | 140  (122-161) | | | 134  (114-160) | | 131  (116-148) | | 128  (108-151) | |
| Oxygen Saturation | 97  (94-99) | 96-97 | | 96  (93-98) | | 96  (93-98) | | 97  (94-99) | 98  (96-99) | | 97  (95-99) | | | 96  (90-98) | | 98  (96-99) | | 97  (95-99) | |
| Temperature | 98.1  (97.5-98.8) | 98.0-98.2 | | 98.6  (97.9-99.9) | | 97.9  (97.4-98.4) | | 99.1  (98-101.1) | 98  (97.4-98.5) | | 97.9  (97.3-98.4) | | | 98.1  (97.4-99.0) | | 98  (97.5-98.5) | | 98  (97.5-98.5) | |
|  |  |  | |  | |  | |  |  | |  | | |  | |  | |  | |
| Critical Vital signs, % |  |  | |  | |  | |  |  | |  | | |  | |  | |  | |
| Tachycardia | 33.5 | 31.1-35.7 | | 43.1 | | 28.4 | | 55.8 | 17.4 | | 25.0 | | | 55.3 | | 25.7 | | 30.1 | |
| Hypotension | 3.8 | 2.3-4.7 | | 2.0 | | 2.2 | | 12.8 | 1.4 | | 4.4 | | | 7.6 | | 3.7 | | 8.6 | |

**Table 3. Subpopulation analyses – High risk diagnoses: Summary demographic statistics of patients included in NHAMCS, 2001-2018 (Weighted N=95,264,481; 5.5% of total)**

*Prior to 2005, entries only allowed for one payer type. For 2005 and beyond, patients may have multiple payer types.

**Reflects data from 2003-2018 as the measure was not collected in 2001 and 2002.

**Figure 3. Subpopulation analyses – High risk diagnoses: Annual trends in vital sign abnormalities^*^**


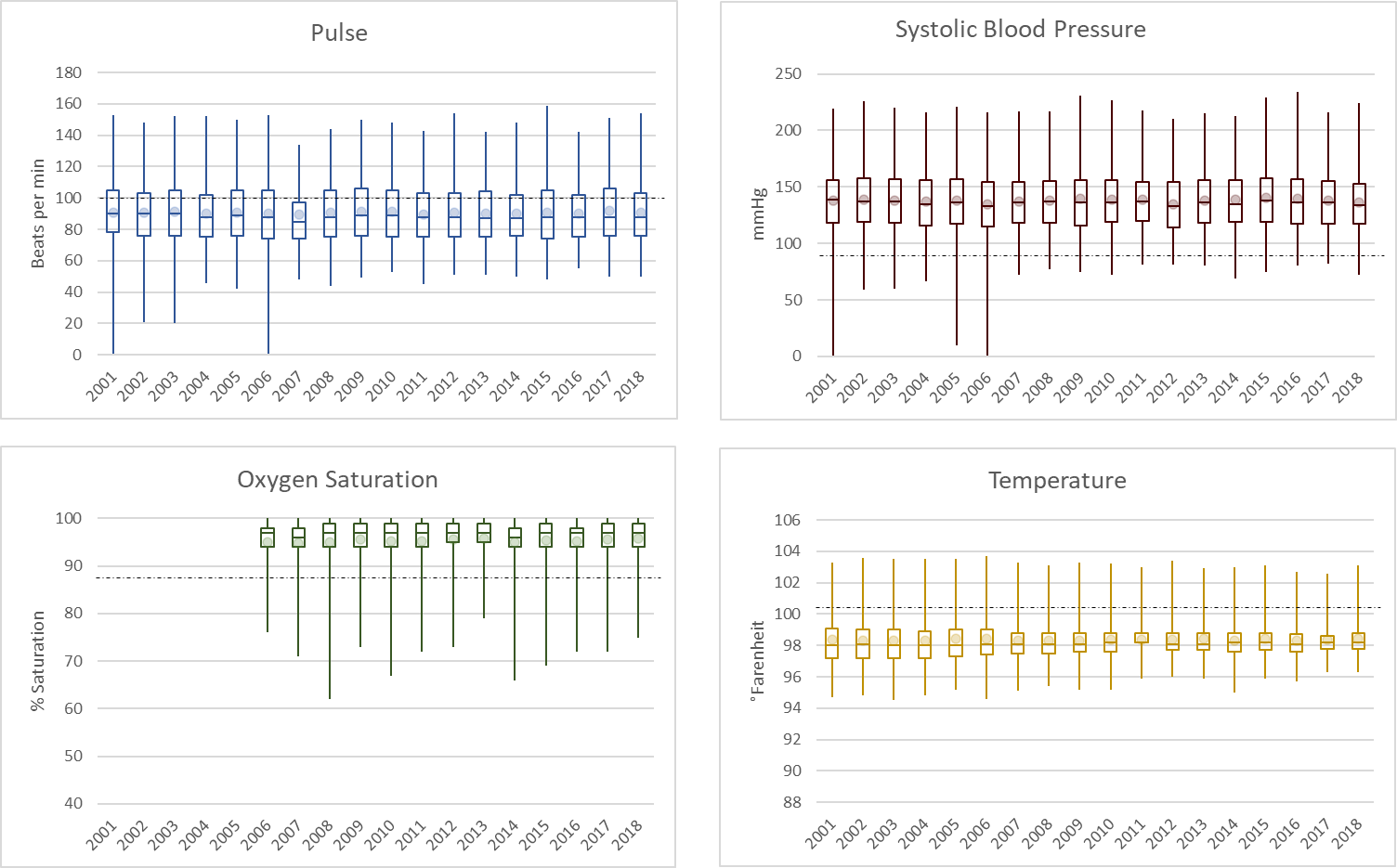


*Box & whisker plot intervals represent 1^st^, 25^th^, 50^th^, 75^th^, and 99^th^ percentiles. Circle overlying box & whisker plot represents annual mean.

**Reference lines have been demarcated for Pulse=100; Systolic blood pressure=90; Oxygen Saturation=88%; Temperature=100.4

**Figure 4. Subpopulation analyses – High risk diagnoses: Annual trends in proportion of patients presenting with tachycardia or hypotension, % (95% CI)**

**
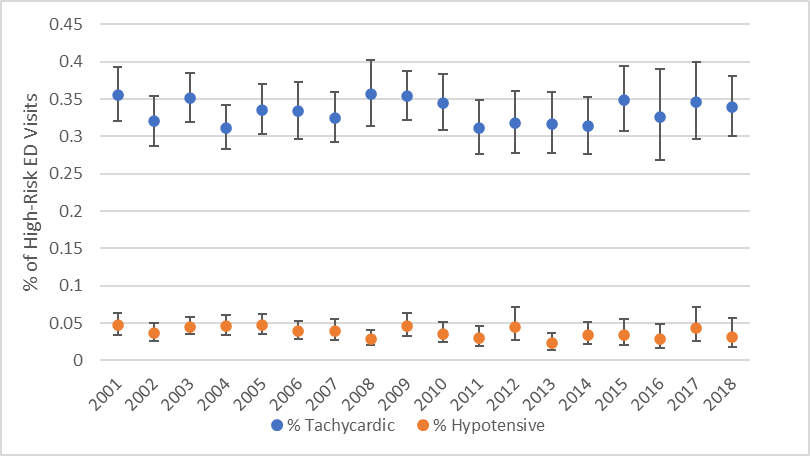
**

**Table 4. Central estimates and 95% CI for patients presented to the ED with tachycardia and hypotension – High risk diagnoses.**

|  | % Tachycardic | 95% CI | | % Hypotensive | 95% CI | |
| --- | --- | --- | --- | --- | --- | --- |
| 2001 | 35.6 | 32.1, | 39.3 | 4.7 | 3.4, | 6.4 |
| 2002 | 32.0 | 28.7, | 35.5 | 3.6 | 2.6, | 5.0 |
| 2003 | 35.2 | 31.9, | 38.5 | 4.5 | 3.5, | 5.8 |
| 2004 | 31.1 | 28.3, | 34.1 | 4.6 | 3.5, | 6.0 |
| 2005 | 33.6 | 30.4, | 37.0 | 4.7 | 3.6, | 6.2 |
| 2006 | 33.4 | 29.6, | 37.3 | 3.9 | 2.8, | 5.3 |
| 2007 | 32.5 | 29.3, | 35.9 | 3.9 | 2.8, | 5.5 |
| 2008 | 35.7 | 31.4, | 40.2 | 2.9 | 2.1, | 4.1 |
| 2009 | 35.4 | 32.1, | 38.8 | 4.6 | 3.3, | 6.4 |
| 2010 | 34.5 | 30.9, | 38.3 | 3.6 | 2.5, | 5.1 |
| 2011 | 31.1 | 27.6, | 34.9 | 3.0 | 1.9, | 4.6 |
| 2012 | 31.8 | 27.8, | 36.0 | 4.5 | 2.7, | 7.2 |
| 2013 | 31.7 | 27.7, | 35.9 | 2.3 | 1.4, | 3.7 |
| 2014 | 31.3 | 27.6, | 35.3 | 3.4 | 2.3, | 5.1 |
| 2015 | 34.9 | 30.7, | 39.4 | 3.4 | 2.1, | 5.6 |
| 2016 | 32.7 | 26.9, | 39.0 | 2.8 | 1.6, | 4.9 |
| 2017 | 34.6 | 29.7, | 39.9 | 4.4 | 2.7, | 7.1 |
| 2018 | 34.0 | 30.1, | 38.1 | 3.2 | 1.8, | 5.7 |

**Table 5. Subpopulation analyses – Age greater than 65 years: Summary demographic statistics of patients included in NHAMCS, 2001-2018 (Weighted N=** **349,469,732; 20.0% of total)**

| Demographics |  |  |
| --- | --- | --- |
|  | **Overall** | **Yearly Range** |
|  |  |  |
| Age, MEDian (iqr) | 76 (70-83) | 75-77 |
| Male, % | 41.6 | 39.9-44.3 |
| Payer type*, % |  |  |
| *Private* | 36.4 | 11.1-48.4 |
| *Medicare* | 77.7 | 70.7-82.1 |
| *Medicaid* | 11.9 | 4.3-15.2 |
| *Self-pay* | 2.9 | 1.5-4.3 |
|  |  |  |
| AMBULANCE Arrival**, % | 36.5 | 32.6-40.7 |
| High risk diagnoses, % | 14.8 | 12.5-18.5 |
|  |  |  |
|  |  |  |
| Vital signs, Median (IQR) |  |  |
| Heart Rate | 80 (70-93) | 79-82 |
| Systolic blood pressure | 144 (126-162) | 141-146 |
| Oxygen Saturation | 91 (95-98) | 97-97 |
| Temperature | 98 (97.5-98.5) | 98.0-98.1 |
|  |  |  |
|  |  |  |
| Critical Vital signs, % |  |  |
| Tachycardia | 19.8 | 17.1-23.2 |
| Hypotension | 1.8 | 0.9-2.8 |

*Prior to 2005, entries only allowed for one payer type. For 2005 and beyond, patients may have multiple payer types.

**Reflects data from 2003-2018 as the measure was not collected in 2001 and 2002.

**Figure 5. Subpopulation analyses – Age greater than 65 years: Annual trends in vital sign abnormalities^*^**

**^
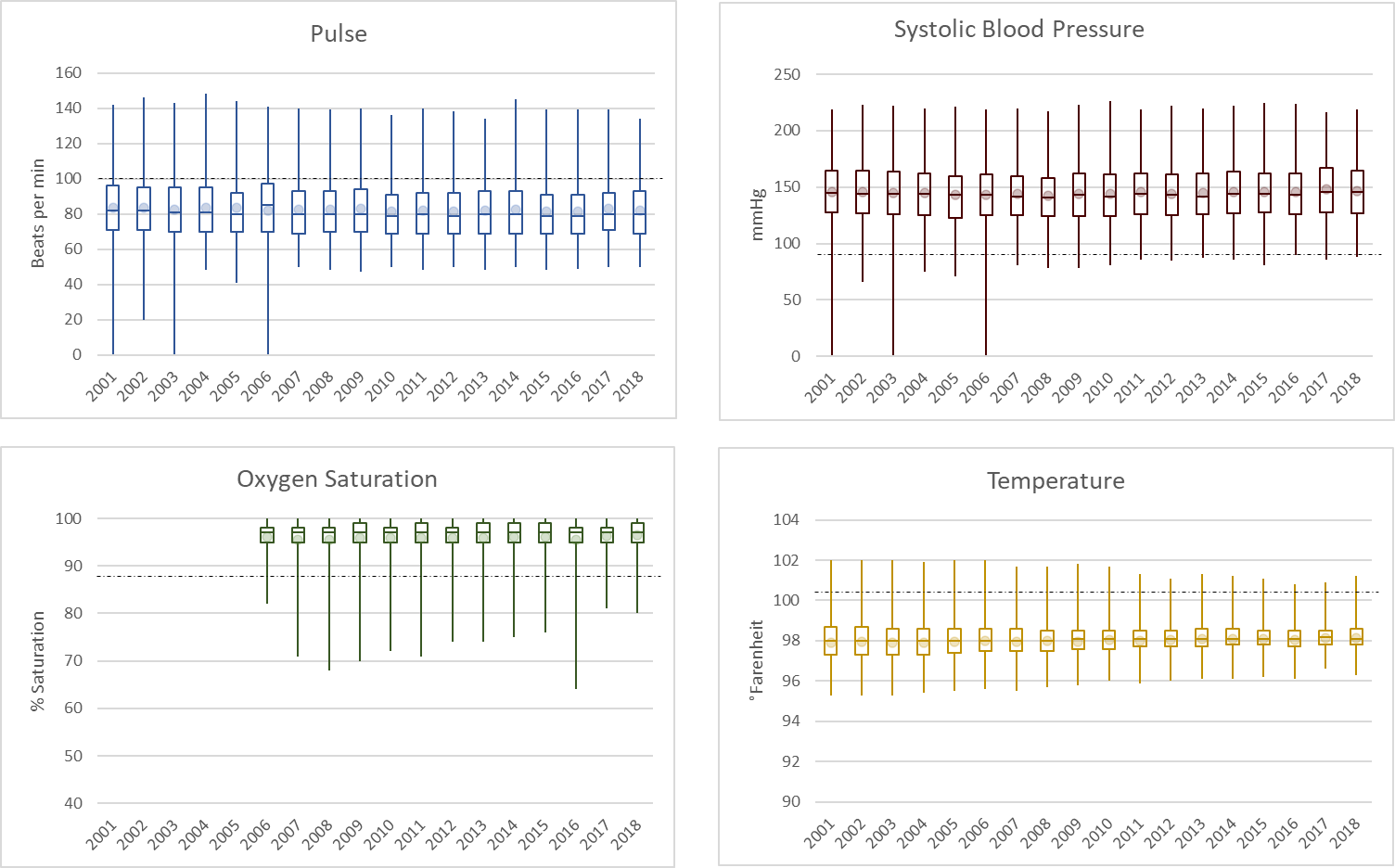
^**

*Box & whisker plot intervals represent 1^st^, 25^th^, 50^th^, 75^th^, and 99^th^ percentiles. Circle overlying box & whisker plot represents annual mean.

**Reference lines have been demarcated for Pulse=100; Systolic blood pressure=90; Oxygen Saturation=88%; Temperature=100.4

**Figure 6. Subpopulation analyses – Age greater than 65 years: Annual trends in proportion of patients presenting with tachycardia or hypotension, % (95% CI)**

**
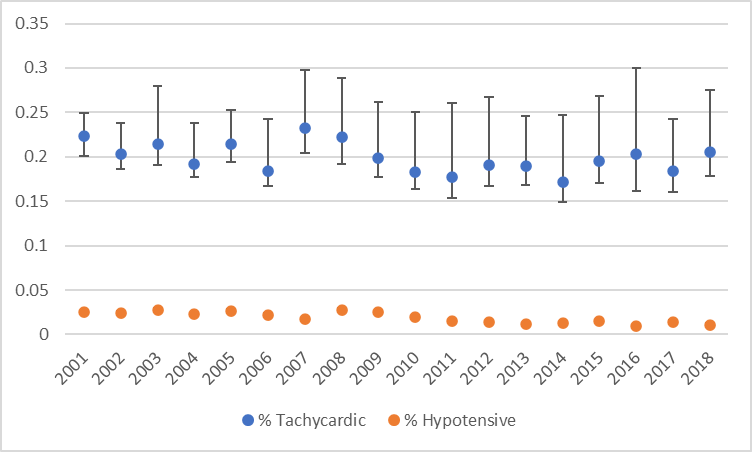
**

*Error bars on hypotension unable to be displayed graphically due to narrow margins.

**Table 6. Central estimates and 95% CI for patients presented to the ED with tachycardia and hypotension – Age greater than 65 years.**

|  | % Tachycardic | 95% CI | | % Hypotensive | 95% CI | |
| --- | --- | --- | --- | --- | --- | --- |
| 2001 | 22.4 | 20.1, | 24.9 | 2.5 | 2.0, | 3.1 |
| 2002 | 20.4 | 18.6, | 23.8 | 2.4 | 2.0, | 2.9 |
| 2003 | 21.5 | 19.1, | 28.0 | 2.8 | 2.3, | 3.4 |
| 2004 | 19.1 | 17.8, | 23.8 | 2.3 | 1.9, | 2.8 |
| 2005 | 21.5 | 19.4, | 25.3 | 2.6 | 2.1, | 3.3 |
| 2006 | 18.4 | 16.7, | 24.3 | 2.2 | 1.7, | 2.9 |
| 2007 | 23.2 | 20.4, | 29.7 | 1.7 | 1.3, | 2.3 |
| 2008 | 22.3 | 19.2, | 28.9 | 2.8 | 2.3, | 3.4 |
| 2009 | 19.9 | 17.7, | 26.2 | 2.5 | 2.0, | 3.1 |
| 2010 | 18.3 | 16.4, | 25.1 | 1.9 | 1.5, | 2.5 |
| 2011 | 17.7 | 15.4, | 26.1 | 1.5 | 1.1, | 2.0 |
| 2012 | 19.1 | 16.7, | 26.7 | 1.4 | 1.0, | 1.9 |
| 2013 | 19.0 | 16.9, | 24.6 | 1.2 | 0.9, | 1.6 |
| 2014 | 17.2 | 14.9, | 24.7 | 1.2 | 0.9, | 1.7 |
| 2015 | 19.6 | 17.0, | 26.8 | 1.5 | 1.0, | 2.3 |
| 2016 | 20.3 | 16.1, | 29.9 | 0.9 | 0.6, | 1.5 |
| 2017 | 18.4 | 16.0, | 24.2 | 1.4 | 0.9, | 2.3 |
| 2018 | 20.6 | 17.9, | 27.5 | 1.1 | 0.7, | 1.7 |

**Table 7. Subpopulation analyses – Patients arriving by ambulance: Summary demographic statistics of patients included in NHAMCS, 2003-2018*. (Weighted N=** **287,694,099; 16.5% of total)**

| Demographics |  |  |
| --- | --- | --- |
|  | **Overall** | **Yearly Range** |
|  |  |  |
| Age, MEDian (iqr) | 56 (39-75) | 54-59 |
| Male, % | 45.9 | 43.6-48.2 |
| Payer type**, % |  |  |
| *Private* | 32.5 | 22.3-374 |
| *Medicare* | 39.5 | 32.2-44.6 |
| *Medicaid* | 24.8 | 13.2-31.6 |
| *Self-pay* | 11.9 | 7.3-15.0 |
|  |  |  |
| High risk diagnoses, % | 12.0 | 8.9-15.0 |
|  |  |  |
|  |  |  |
| Vital signs, Median (IQR) |  |  |
| Heart Rate | 85 (73-98) | 84-86 |
| Systolic blood pressure | 135 (119-152) | 133-136 |
| Oxygen Saturation | 98 (96-99) | 98-98 |
| Temperature | 98.1 (97.6-98.6) | 97.9-98.2 |
|  |  |  |
|  |  |  |
| Critical Vital signs, % |  |  |
| Tachycardia | 24.6 | 22.8-29.9 |
| Hypotension | 2.1 | 1.4-3.7 |

*Reflects data from 2003-2018 as the measure was not collected in 2001 and 2002.

**Prior to 2005, entries only allowed for one payer type. For 2005 and beyond, patients may have multiple payer types.

**Figure 7. Subpopulation analyses – Patients arriving by ambulance: Annual trends in vital sign abnormalities^*^**

**^
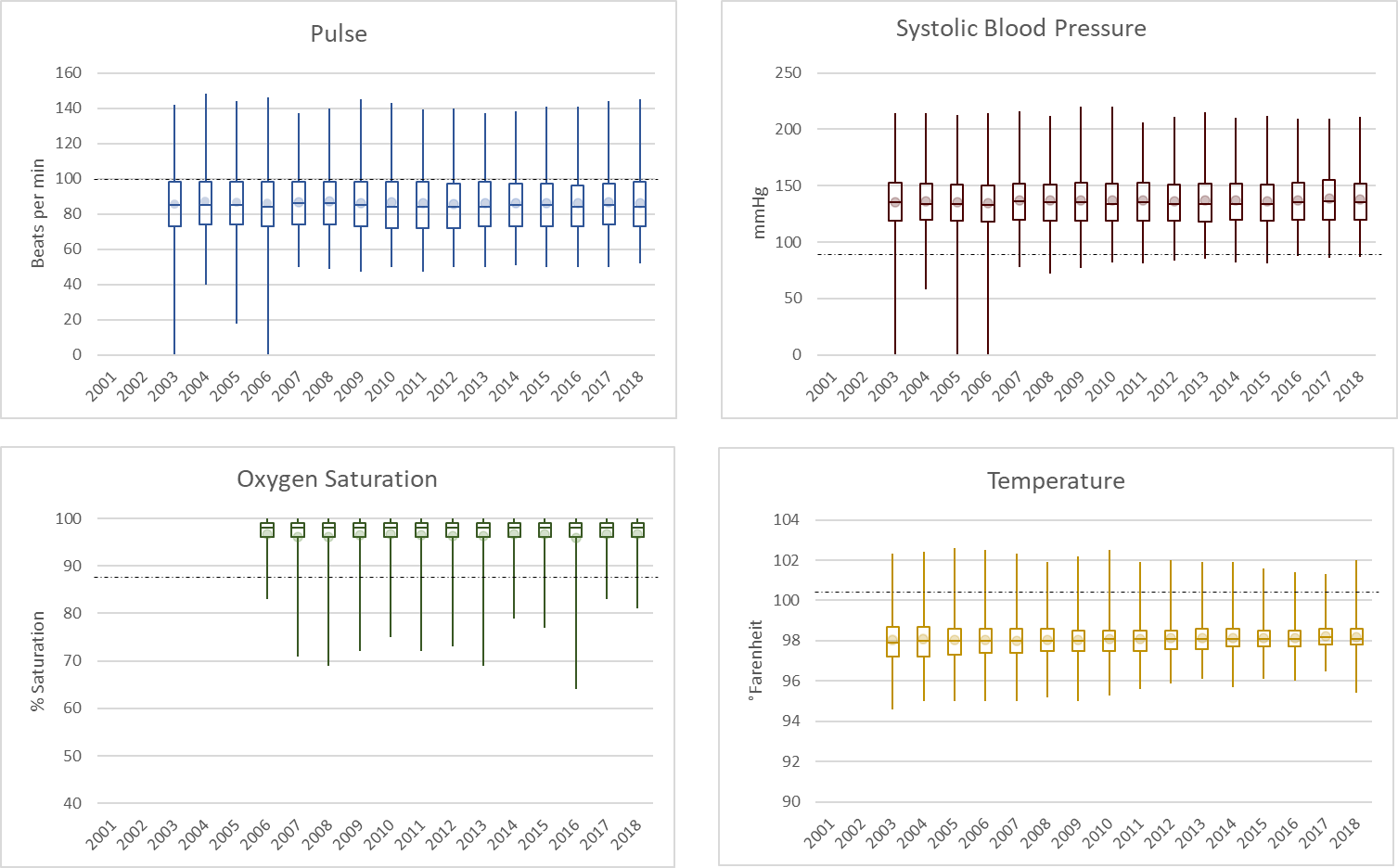
^**

*Box & whisker plot intervals represent 1^st^, 25^th^, 50^th^, 75^th^, and 99^th^ percentiles. Circle overlying box & whisker plot represents annual mean.

**Reference lines have been demarcated for Pulse=100; Systolic blood pressure=90; Oxygen Saturation=88%; Temperature=100.4

**Figure 8. Subpopulation analyses – Patients arriving by ambulance: Annual trends in proportion of patients presenting with tachycardia or hypotension, % (95% CI)**

*Error bars on hypotension unable to be displayed graphically due to narrow margins.

**Table 8. Central estimates and 95% CI for patients presented to the ED with tachycardia and hypotension – Patients arriving by ambulance.**

|  | % Tachycardic | 95% CI | | % Hypotensive | 95% CI | |
| --- | --- | --- | --- | --- | --- | --- |
| 2001 | **-** |  |  | **-** |  |  |
| 2002 | **-** |  |  | **-** |  |  |
| 2003 | 25.6 | 23.4, | 27.9 | 3.7 | 3.1, | 4.3 |
| 2004 | 24.4 | 22.9, | 26.1 | 2.7 | 2.2, | 3.3 |
| 2005 | 24.4 | 22.4, | 26.5 | 2.9 | 2.4, | 3.6 |
| 2006 | 24.2 | 22.4, | 26.1 | 2.7 | 2.2, | 3.4 |
| 2007 | 29.9 | 26.0, | 34.0 | 2.1 | 1.7, | 2.7 |
| 2008 | 27.6 | 24.5, | 31.0 | 2.4 | 1.9, | 3.1 |
| 2009 | 24.4 | 22.2, | 26.6 | 2.5 | 2.0, | 3.0 |
| 2010 | 25.9 | 23.9, | 27.9 | 1.8 | 1.4, | 2.4 |
| 2011 | 25.0 | 22.5, | 27.7 | 2.0 | 1.5, | 2.6 |
| 2012 | 24.7 | 22.3, | 27.3 | 1.8 | 1.3, | 2.6 |
| 2013 | 25.6 | 22.5, | 28.9 | 1.6 | 1.1, | 2.2 |
| 2014 | 24.1 | 20.1, | 28.6 | 1.4 | 1.0, | 2.1 |
| 2015 | 26.4 | 22.9, | 30.2 | 1.7 | 1.1, | 2.5 |
| 2016 | 29.2 | 22.6, | 36.7 | 1.0 | 0.7, | 1.5 |
| 2017 | 22.8 | 20.0, | 25.9 | 2.0 | 1.3, | 3.0 |
| 2018 | 25.7 | 22.9, | 28.8 | 1.4 | 0.9, | 2.2 |

**Table 9. Subpopulation analyses – Publicly insured patients: Summary demographic statistics of patients included in NHAMCS, 2001-2018. (Weighted N=** **730,347,091; 41.8% of total)**

| Demographics |  |  |
| --- | --- | --- |
|  | **Overall** | **Yearly Range** |
|  |  |  |
| Age, MEDian (iqr) | 55 (34-74) | 52-64 |
| Male, % | 38.3 | 36.1-40.3 |
|  |  |  |
| AMBULANCE Arrival**, % | 25.5 | 21.8-29.8 |
| High risk diagnoses, % | 8.5 | 6.1-11.7 |
|  |  |  |
|  |  |  |
| Vital signs, Median (IQR) |  |  |
| Heart Rate | 85 (74-97) | 84-85 |
| Systolic blood pressure | 135 (120-152) | 134-137 |
| Oxygen Saturation | 98 (96-99) | 98-98 |
| Temperature | 98.1 (97.6-98.6) | 97.9-98.2 |
|  |  |  |
|  |  |  |
| Critical Vital signs, % |  |  |
| Tachycardia | 23.5 | 21.6-26.9 |
| Hypotension | 1.2 | 0.6-1.9 |

*Prior to 2005, entries only allowed for one payer type. For 2005 and beyond, patients may have multiple payer types.

**Reflects data from 2003-2018 as the measure was not collected in 2001 and 2002.

**Figure 8. Subpopulation analyses – Publicly insured patients: Annual trends in vital sign abnormalities^*†^**


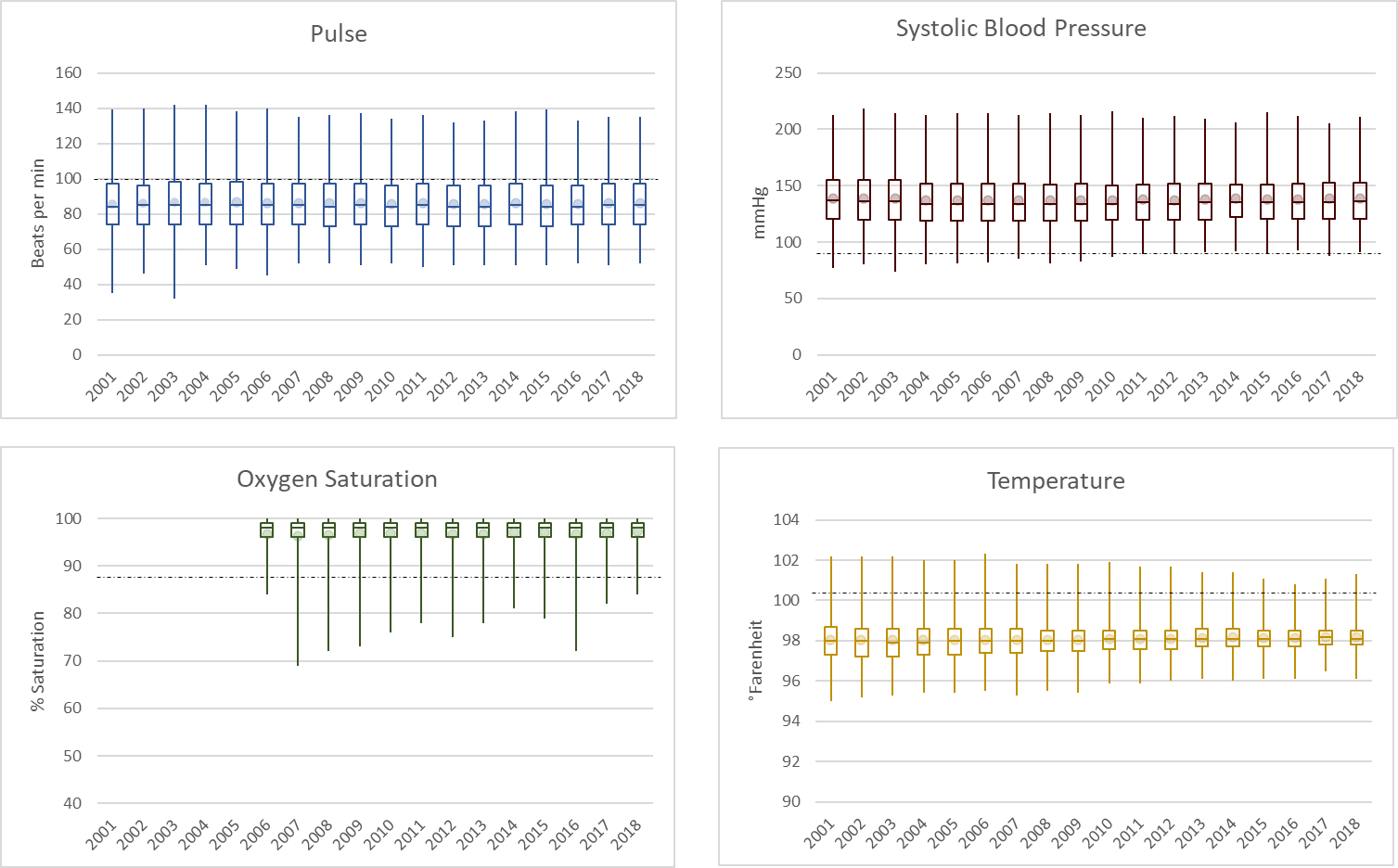


*Box & whisker plot intervals represent 1^st^, 25^th^, 50^th^, 75^th^, and 99^th^ percentiles. Circle overlying box & whisker plot represents annual mean.

**Reference lines have been demarcated for Pulse=100; Systolic blood pressure=90; Oxygen Saturation=88%; Temperature=100.4

**Figure 9. Subpopulation analyses – Publicly insured patients: Annual trends in proportion of patients presenting with tachycardia or hypotension, % (95% CI)**

**
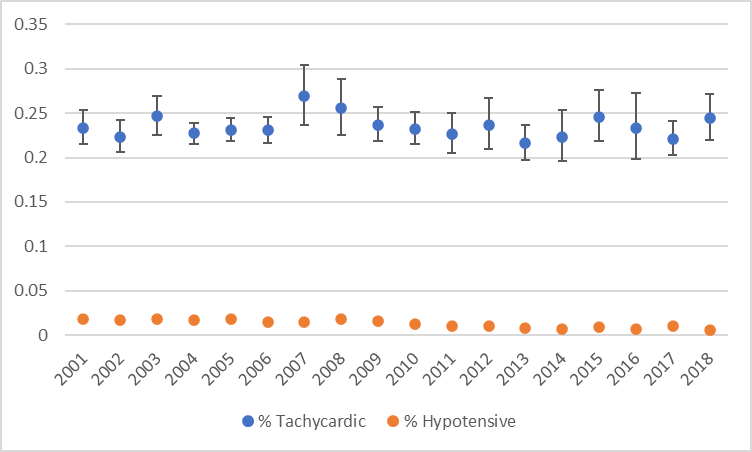
**

*Error bars on hypotension unable to be displayed graphically due to narrow margins.

**Table 10. Central estimates and 95% CI for patients presented to the ED with tachycardia and hypotension – Publicly insured patients.**

|  | % Tachycardic | 95% CI | | % Hypotensive | 95% CI | |
| --- | --- | --- | --- | --- | --- | --- |
| 2001 | 23.4 | 21.5, | 25.3 | 1.9 | 1.5, | 2.2 |
| 2002 | 22.3 | 20.6, | 24.2 | 1.7 | 1.4, | 2.1 |
| 2003 | 24.7 | 22.5, | 26.9 | 1.9 | 1.6, | 2.2 |
| 2004 | 22.7 | 21.5, | 23.9 | 1.8 | 1.5, | 2.1 |
| 2005 | 23.1 | 21.9, | 24.4 | 1.8 | 1.5, | 2.2 |
| 2006 | 23.1 | 21.6, | 24.6 | 1.5 | 1.2, | 1.9 |
| 2007 | 26.9 | 23.6, | 30.4 | 1.5 | 1.2, | 1.8 |
| 2008 | 25.6 | 22.6, | 28.8 | 1.9 | 1.5, | 2.2 |
| 2009 | 23.7 | 21.8, | 25.7 | 1.6 | 1.4, | 1.9 |
| 2010 | 23.2 | 21.5, | 25.1 | 1.3 | 1.1, | 1.6 |
| 2011 | 22.7 | 20.5, | 25.0 | 1.0 | 0.8, | 1.3 |
| 2012 | 23.7 | 20.9, | 26.7 | 1.0 | 0.7, | 1.4 |
| 2013 | 21.6 | 19.7, | 23.7 | 0.8 | 0.6, | 1.1 |
| 2014 | 22.3 | 19.6, | 25.4 | 0.7 | 0.5, | 0.9 |
| 2015 | 24.6 | 21.9, | 27.6 | 0.9 | 0.7, | 1.3 |
| 2016 | 23.3 | 19.8, | 27.2 | 0.7 | 0.5, | 1.0 |
| 2017 | 22.1 | 20.2, | 24.1 | 1.1 | 0.8, | 1.4 |
| 2018 | 24.5 | 22.0, | 27.2 | 0.6 | 0.5, | 0.9 |
